# AICAR Improves Outcomes of Metabolic Syndrome and Type 2 Diabetes Induced by High-Fat Diet in C57Bl/6 Male Mice

**DOI:** 10.3390/ijms232415719

**Published:** 2022-12-11

**Authors:** Elena A. Tukhovskaya, Elvira R. Shaykhutdinova, Irina A. Pakhomova, Gulsara A. Slashcheva, Natalya A. Goryacheva, Elena S. Sadovnikova, Ekaterina A. Rasskazova, Vitaly A. Kazakov, Igor A. Dyachenko, Alina A. Frolova, Alexey N. Brovkin, Vasiliy E. Kaluzhsky, Mikhail Yu. Beburov, Arkady N. Murashev

**Affiliations:** 1 Biological Testing Laboratory, Branch of Shemyakin and Ovchinnikov Institute of Bioorganic Chemistry, Russian Academy of Sciences, Prospekt Nauki, 6, Pushchino, 142290 Moscow, Russia; 2 LLC “OKA-BIOTECH”, Novatorov St., d. 34, bldg. 7, apt. 42, 119421 Moscow, Russia

**Keywords:** metabolic syndrome, type 2 diabetes, obesity, C57BL/6 mice

## Abstract

The aim of the study was to investigate the effect of AMP-activated protein kinase activator 5-aminoimidazole-4-carboxamide ribonucleoside (AICAR) on the consequences of metabolic syndrome and type 2 diabetes induced by the consumption of a high-fat diet (HFD) in male C57Bl/6 mice. Additionally, the animals from group 6 were administered Methotrexate (MTX) at a dose of 1 mg/kg in parallel with AICAR, which slows down the metabolism of AICAR. The animals were recorded with signs of metabolic syndrome and type 2 diabetes mellitus by recording their body weights, glucose and insulin levels, and the calculating HOMA-IRs. At the end of the study, at the end of the 13th week, during necropsy, the internal organs were assessed, the masses of the organs were recorded, and special attention was paid to visceral fat, assessing its amount and the mass of the fat surrounding epididymis. The biochemical parameters and histology of the internal organs and tissues were assessed. The animals showed signs of metabolic syndrome and type 2 diabetes, namely, weight gain, hyperglycemia, hyperinsulinemia, an increase in the amount and mass of abdominal fat, and metabolic disorders, all expressed in a pathological change in biochemical parameters and pathological changes in internal organs. The AICAR treatment led to a decrease in body weight, a decrease in the amount and mass of abdominal fat, and an improvement in the pathomorphological picture of internal organs. However, some hepatotoxic effects were observed when the animals, on a received standard diet (STD), were treated with AICAR starting from the first day of the study. The additional administration of MTX, an AICAR metabolic inhibitor, did not improve its efficacy. Thus, AICAR has therapeutic potential for the treatment of metabolic syndrome and type 2 diabetes.

## 1. Introduction

AICAR is an activator of cellular protein kinase that regulates intracellular energy balance (AMPK) [1]. The therapeutic potential of AICAR is due to the fact that the pathological processes that cause disturbances in the action of AMPK can cause such dangerous conditions as hypertension, ischemia, cancer, Alzheimer’s disease, obesity, and diabetes. AICAR has anti-ischemic properties. Thus, in the work of Leung et al. it was shown that AICAR limits the severity of myocardial ischemia after bypass surgery, as evidenced by the shorter duration of ischemia in patients receiving high doses of AICAR [2]. AICAR receives orphan drug designation for adult chronic lymphocytic leukemia (B-Cell) in the European Union [3,4]. In 2007, it was found that the activation of AMP-activated protein kinase (AMPK) in muscle by AICAR, as with exercise, increases glucose uptake [5]. The course application of AICAR induces a metabolic shift in cardiomyocytes exposed to free fatty acids, characterized by improved glucose transport and the redirection of fatty acids to neutral storage. Such metabolic changes in vivo may protect the heart of patients with type 2 diabetes from ischemia-reperfusion injury [6]. AICAR was found to be able to inhibit the synthesis of fatty acids and cholesterol in liver cells [7], to enhance fatty acid oxidation [8], and inhibit gluconeogenesis [9]. In skeletal muscle cells, AICAR has been found to activate glycogen phosphorylase and increase glycogenolysis [10], stimulate fatty acid oxidation and glucose uptake [11], and inhibit protein synthesis [12]. All of these studies have confirmed the role of AMPK as the central metabolic site of the cell, which is activated whenever the AMP/ATP ratio is high and acts to increase catabolic and inhibit anabolic processes. The previously studied AICAR effects indicate the possibility of its use for the treatment of type 2 diabetes [13]. AICAR induces hypoglycemia in vivo [14,15]. In addition, AICAR may help reduce peripheral insulin resistance [16]. In various animal models of insulin resistance, the administration of AICAR has been shown to improve metabolic disturbances and enhance insulin sensitivity in peripheral tissues [17,18,19,20]. AICAR systemic administration in humans has beneficial effects by reducing hepatic glucose production and increasing skeletal muscle glucose uptake [5,21]. AICAR also has great potential in the treatment of metabolic syndrome associated with obesity. The problem of obesity is now widespread in the world; the complications that develop in this condition include diabetes mellitus, non-alcoholic fatty liver disease, and kidney disease [22,23]. Metabolic syndrome is a constellation of clinical features consisting of abdominal obesity, high glucose, high triglycerides and low HDL cholesterol, and arterial hypertension. The presence of metabolic syndrome entails an increase in the risk of developing cardiovascular diseases and risk of developing type 2 diabetes mellitus. The treatment includes diet and exercise for weight loss, as well as medical treatment for atherogenic dyslipidemia and hyperglycemia [24]. AICAR reduces inflammation in human adipose tissue explants and in mice fed a high-fat diet, promotes weight loss, and also reduces inflammation in adipocytes, while reducing damage to the liver and kidneys, as well as reducing resistance to glucose, which may indicate that it helps to reduce the complications caused by obesity [25]. AICAR reduces glucose levels, which has been shown in genetically modified mice with diabetes and obesity [26]. In order to lower the relatively high AMPK activation threshold [11], as well as increase the relatively low bioavailability [27], limiting the effectiveness of AICAR in the treatment of type 2 diabetes [21], it was decided to administer AICAR in combination with the antitumor drug Methotrexate, which can increase the activation of AMPK stimulated by AICAR in skeletal muscles, to one of the groups of animals [28]. It has previously been shown that Methotrexate significantly lowered the threshold for AICAR-stimulated AMPK activation and also potentiated glucose uptake and lipid oxidation in skeletal muscle [28].

The aim of the study was to evaluate the efficacy of AICAR in the metabolic syndrome aggravated by diabetes mellitus in C57Bl/6 mice fed a high-fat diet combined with circadian rhythm disturbance.

## 2. Results

Two animals died in the study. The first animal died in group 6 (HFD + AC + MTX) at week 9 of the study and the second animal died in group 5 (HFD + AC 7) at week 10 of the study. It should be noted that starting from the eighth week of the study, baldness of various parts of the body was observed in animals treated with HFD: chin, neck, head, and front paws. The number of animals with deviations was the highest in group 6 (HFD + AC + MTX 7), probably because hair loss is one of the known side effects of Methotrexate.

### 2.1. Body Weight and Body Weight Gain

The initial body weight of the animals in all groups at the beginning of the study did not differ. By the end of the fourth week of the study (Day 28), there was a significant increase in body weight relative to the control group in groups 3 (HFD + vehicle), 5 (HFD + AC 7), and 6 (HFD + AC + MTX) (26.8 ± 2.0 g, 26.4 ± 1.7 g and 26.7 ± 1.9 g, respectively, versus 24.7 ± 1.1 g in the STD + vehicle group). Starting from the seventh week of the study (42nd day), after the end of the two-week period of keeping the animals under constant illumination designed to disturb the circadian rhythm, the absolute weight of the animals was significantly higher in comparison with the control, the weight of animals from group 4 (HFD + AC 1), 3 (HFD + vehicle), 5 (HFD + AC 7), and 6 (HFD + AC + MTX) (30.6 ± 2.6 g, 29.2 ± 2.1 g, 29.2 ± 2.6 g and 29.5 ± 2.6 g, respectively, versus 26.1 ± 1.0 g in the STD + vehicle group). This difference in weight remained unchanged throughout the lifetime phase of the study, i.e., the absolute weight of all the animals treated with HFD was significantly different from the weight of control animals from the fourth week of the study (Table 1). The same differences were observed when comparing the parameter “weight gain” (Table 2). Assessing the body weight of animals treated with AICAR against the background of HFD starting from the seventh week, it was found that AICAR alone (from the tenth week), as well as in combination with MTX (starting from the ninth week), reduces the absolute body weight relative to the animals from group 3 receiving only HFD (Table 3). At the same time, the body weight, as well as the increase in body weight of animals from group 4, which received AICAR against the background of HFD starting from the first day, did not differ from that in group 3, which received HFD and the vehicle (Table 1 and Table 2). In group 5 (HFD + AC 7), at week 11, there was a significant decrease in body weight gain relative to group 3 (HFD + vehicle) (38.6 ± 12.7% versus 52.0 ± 14.9%), which indicates that that AICAR treatment from seventh week of the study, helps to reduce body weight gain. Thus, summarizing the data on body weight and body weight gain, we can conclude that HFD in male C57Bl/6 mice promotes weight gain starting at the fourth week of the study, while AICAR, administered starting from the seventh week of the study, either alone or in combination with MTX, resulted in a decrease in absolute body weight starting at weeks 10 and 9 of the study compared to animals treated with HFD alone. The rate of weight gain in animals treated with HFD was significantly increased relative to the control starting from the fifth week of the study. This increase was maintained throughout the study in all animals on HFD, with the exception of the group 5 animals treated with AICAR from week 7 of the study. In general, it can be concluded that AICAR, administered starting from the seventh week of the study, contributes to the reduction in absolute body weight and weight gain in animals receiving HFD.

### 2.2. Food Consumption

Food consumption varied from the first week of the study. As early as day 7, the food intake in all HFD-treated groups was reduced compared to STD-treated groups (Table 4). These differences persisted until the end of the study. In group 4, treated with HFD + AC 1, on the 35th day of the study (week 5), food intake was significantly higher compared to animals from group 3 (HFD + vehicle). In group 5 (HFD + AC 7), food intake was higher compared to group 3 (HFD + vehicle) on the 49th day (seventh week) (Table 4). Additionally, intragroup differences were observed in all the groups relative to the 7th and 21st days of the study, except for group 4 (HFD + AC 1) (Table 2). Starting from the fifth (groups 1, 2, 3, 6) week or from the seventh (group 2) week, the animals showed an increase in food consumption relative to the first week of the study. The exception was the animals in Group 4 (HFD + AC 1)—in these animals, there were no intragroup differences in food consumption during the entire observation period. Thus, it can be concluded that HFD resulted in a reduction in food consumption from week 1 of the study in all HFD-treated groups. In all animals, with the exception of animals treated with AICAR starting from the first day of the study, the food consumption increased from the fifth or seventh week.

### 2.3. Insulin Resistance Test

Baseline glucose values were measured in the animals on empty stomachs before insulin administration, as well as in dynamics 20, 40, 60, and 120 min after subcutaneous administration of insulin at a dose of 2 IU/kg. The results of the experiment are presented in Table 5. In C57BL/6 mice kept on HFD, the baseline hyperglycemia was recorded—the initial blood glucose levels in groups 3, 4, 5, and 6 were significantly higher relative to animals from group 1 kept on a standard diet (STD + vehicle) and group 2 (STD + AC). The introduction of insulin significantly reduced the level of glucose in the blood from the initial values in each of the groups after 20 min. Hypoglycemia compared with the baseline values persisted until the end of the experiment. In this experiment, all the animals showed sensitivity to insulin, since the glucose level was significantly reduced in all animals (Table 3).

### 2.4. Oral Glucose Tolerance Test

All the animals after an overnight fast were measured for the initial glucose level, after which a 40% glucose solution at a dose of 2 mg/kg was injected into the stomach with a probe and the amount of glucose was measured 30, 60, 90, and 120 min after administration. Table 6 presents the average values of changes in blood glucose levels in the animals. As noted above, initially, all the animals treated with HFD had elevated glucose levels (7.4 ± 1.5 mmol/L in group 3, 7.9 ± 1.8 mmol/L in group 4, 8.4 ± 0.6 mmol/L in group 5, 7.4 ± 0.9 mmol/L in group 6 vs. 5.2 ± 0.4 mmol/L in group 1 and 6.2 ± 0.9 mmol/L in group 2) (Table 3). After glucose administration to all the mice, hyperglycemia was observed after 30 min (relative to baseline values). Significantly elevated glucose concentrations persisted until the 120th minute in groups 2 (STD + AC), 3 (HFD + vehicle), 5 (HFD + AC 7), and 6 (HFD + AC + MTX), while in groups 1 (STD + vehicle) and 4 (HFD + AC 1) the glucose levels did not differ from the baseline by 120 min. In all the groups, the maximum glucose level was observed by the 30th minute and, from the 60th minute to the 120th minute, the glucose values were significantly reduced relative to this maximum value (Table 7). At the same time, only in groups 1 (STD + vehicle) and 4 (HFD + AC 1) did the values fully recover to the initial level by the 120th minute. So, in all the animals treated with HFD, as well as in the animals treated with AICAR on the background of a standard diet, glucose tolerance was observed. The animals treated with AICAR from week 7 alone (group 5) or in combination with Methotrexate (group 6) did not improve the glucose tolerance. Some improvement in glucose tolerance can be seen in the case of AICAR against the background of HFD from the first day of the study (group 4) (Table 6).

### 2.5. Insulin Resistance Assessment—HOMA-IR

**Figure 1:**
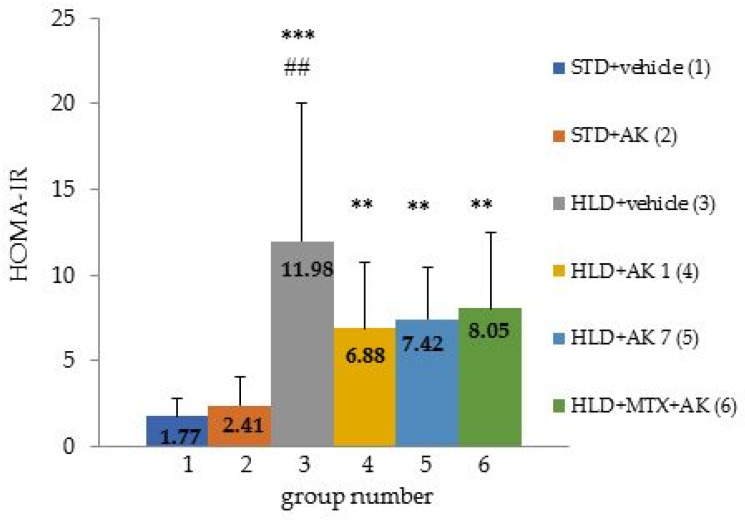
HOMA-IR in C57Bl/6 mice fed an STD (Groups 1, 2) or a HFD (Groups 3, 4, 5, 6). Data are presented as group mean + standard deviation. ** *p* ≤ 0.01, *** *p* ≤ 0.001 relative to group 1 (STD + vehicle) according to Kruskal–Wallis ANOVA, ## *p* ≤ 0.01 relative to group 2 (STD + AC) according to Kruskal–Wallis ANOVA.

To assess insulin resistance in all the animals, the homeostasis model assessment of insulin resistance index (HOMA-IR) was calculated. Blood was extracted from the animals after an overnight fast on the day of necropsy. The HOMA-IR index was calculated as follows: fasting glucose level mmol/L x fasting insulin level (mIU/L)/22.5. It was shown that in all the animals kept on an HFD, the HOMA-IR index was increased relative to the animals kept on an STD (11.98 ± 8.07 in group 3, 6.88 ± 3.87 in group 4, 7.42 ± 3.05 in group 5, and 8.05 ± 4.4 in group 6 versus 1.77 ± 1.61 in group 1) (Figure 1). That is, the animals kept on HFD developed insulin resistance, indicating the development of metabolic syndrome, as well as type 2 diabetes. Visually (Figure 1), the AICAR-treated animals had a lower HOMA-IR compared to the group 3 animals kept on HFD and untreated. The fasting insulin and glucose values are shown in Table 5. The insulin level was measured after an overnight fast using an enzyme-linked immunosorbent assay in the serum of all the animals in the blood extracted during necropsy from the inferior vena cava. In group 1 (STD + vehicle), the hormone concentration was 6.23 mIU/L. When keeping mice on HFD, insulin levels increased several times. So, in groups 3, 5, and 6, insulin was significantly increased compared to group 1, with the exception of group 4 (HFD + AC 1), in which the hormone level did not statistically differ from group 1. However, if we compare groups on HFD with each other, then a significant decrease in the indicator in the AICAR-treated groups from the first or seventh week of the study (groups 4 and 5, respectively) is clearly visible: insulin significantly decreased relative to group 3 (HFD + vehicle). Thus, when C57BL mice are kept on an HFD, in addition to weight gain and hyperglycemia, hyperinsulinemia is detected, which confirms the presence of diabetes mellitus in animals. The AICAR treatment from the first week of the study was most effective in reducing fasting insulin levels—the insulin levels in group 4 did not significantly differ from group 1, which is on STD.

### 2.6. Assessment of Locomotor Activity

The average values of the indicators of the motor activity of the animals according to the test “Open field” performed using the TSE Multi Conditioning System Extended Advanced are presented in Table 8. The distance traveled in the center by the animals treated with HFD (except animals from group 4 (HFD + AC 1)) significantly decreased (2 ± 1 m in group 3, 2 ± 1 m in group 5 and 2 ± 2 m in group 6 versus 5 ± 1 m in group 2). The decrease in the distance traveled in the center by the animals treated with HFD indirectly indicates a higher level of anxiety compared to the animals kept on an STD. The absence of a difference in the distance traveled in the center from the animals treated with STD in the animals treated with HFD + AC 1 indirectly indicates some protective function of AICAR in relation to HFD-depending anxiety.

### 2.7. Blood Chemistry

As can be seen from Table 7, there were significant changes in basic blood parameters in the animals kept on a high-fat diet. For example, the urea concentrations were significantly lower in Groups 3–6 compared to Group 1 (6.9 ± 0.7 mmol/L, 6.7 ± 1.0 mmol/L, 6.0 ± 0.5 mmol/L and 6.2 ± 0.9 mmol/L vs. 10.7 ± 0.8 mmol/L, respectively). There were also differences in the concentration of blood lipid components: triglycerides (unreliable) and cholesterol. Thus, the value of total cholesterol in the biochemical analysis of the animal blood of the groups kept on HFD was significantly higher than the values of the groups on STD by almost 2 times (5.50 ± 0.69 mmol/L, 4.67 ± 0.52 mmol/L, 4.71 ± 0.38 mmol/L and 4.53 ± 0.43 mmol/L vs. 2.68 ± 0.34 mmol/L, respectively). When comparing groups 3, 4, 5, and 6, kept on HFD, and group 1, kept on an STD, in addition to a decrease in urea and an increase in cholesterol, the former showed a significant decrease in ALT levels (37 ± 7 U/L, 35 ± 10 U/L, 35 ± 7 U/L and 31 ± 4 U/L vs. 63 ± 8 U/L, respectively), AST (significantly only in group 6—53 ± 4 U/L vs. 67 ± 6 U/L in group 1), alkaline phosphatase (54 ± 7 U/L, 54 ± 8 U/L, 54 ± 8 U/L and 50 ± 5 U/L vs. 98 ± 9 U/L, respectively), and the ratio of albumin and globulins (1.6 ± 0.1 in group 3, 1.6 ± 0.1 in group 4 vs. 1.8 ± 0.1 in group 1), due to the tendency to decrease in the albumin level. In the animals kept on HFD, a decrease in the total bilirubin was observed, significantly only in group 4 (5.1 ± 1.8 mmol/L in group 4 vs. 7.6 ± 1.6 mmol/L in group 2). There was a decrease in the level of inorganic phosphates and chlorides in group 6 relative to group 1 (3.28 ± 0.17 mmol/L vs. 3.94 ± 0.53 mmol/Lfor phosphates, 124 ± 2 mmol/L vs. 127.1 ± 3.5 mmol/L for chlorides). The introduction of AICAR against the background of HFD effectively reduced the level of cholesterol relative to group 3 kept on HFD and not receiving treatment (4.67 ± 0.52 mmol/L in group 4, 4.71 ± 0.38 mmol/L in group 5, 4.53 ± 0.43 mmol/L in group 6 vs. 5.50 ± 0.69 mmol/L in group 3).

### 2.8. Necropsy Results

At the planned necropsy of the animals (13th week of the study), macroscopic abnormalities were assessed, which are summarized in Table 9. The number of animals with macroscopic abnormalities from group 3 HFD + vehicle significantly exceeded those in the groups kept on STD. In group 5 HFD + AC 7, there were significantly fewer animals with signs of abnormalities relative to group 3. In all the animals kept on HFD (groups 3–6), visceral obesity of the abdominal organs was visually observed and, in group 3 (HFD + vehicle), this deviation was most pronounced. The introduction of AICAR against the background of HFD in groups 4, 5, and 6 led to a significant decrease in the number of animals with an increased content of fat cells in the abdominal cavity. Additionally, a frequently observed macroscopic abnormality in the HFD groups was hydronephrosis of one of the kidneys (the largest in group 3—in 50% of the animals), which may be a consequence of narrowing of the ureteropelvic junction due to the formation of atherosclerotic vascular lesions and ischemia of the kidney tissue with excessive fat intake.

### 2.9. Organ Weight

The adrenal mass in groups treated with AICAR monocomponent from the seventh week (group 5) and in combination with Methotrexate (group 6) was lower than in the other groups, in particular with group 3 (HFD + vehicle) (0.0038 ± 0 0.0010 g in group 5 and 0.0048 ± 0.0021 g in group 6 vs. 0.0079 ± 0.0011 g in group 3). All the animals treated with HFD had a reduced absolute liver mass relative to the animals from groups 1 and 2 receiving a standard diet (1.37 ± 0.18 g (not significant) in group 3, 1.28 ± 0.19 g in group 4, 1.27 ± 0.10 g in group 5, and 1.28 ± 0.14 g in group 6 vs. 1.59 ± 0.43 g in group 1 and 1.73 ± 0.19 g in group 2). The pancreatic weight was lower in animals from groups 5 (HFD + AC 7—0.118 ± 0.017 g) and 6 (HFD + MTX + AC—0.148 ± 0.047 g) compared with animals receiving a standard diet from groups 1 (0.158 ± 0.047 g). 0.017 g) and 2 (0.220 ± 0.075). Additionally, in all the animals treated with HFD, the mass of adipose tissue surrounding the epididymis was increased relative to animals receiving a standard diet (1.7047 ± 0.4698 g in group 3, 1.0991 ± 0.2389 g in group 4, 1.2541 ± 0.3390 g in group 5, and 1.1489 ± 0.4382 g in group 6 vs. 0.4556 ± 0.0708 g in group 1 and 0.4259 ± 0.0816 g in group 2). The relative organ mass had the same differences as the absolute organ mass (Table 10). Particular attention was paid to the mass of adipose tissue surrounding the epididymis. As an indicator of obesity, we chose the adipose tissue surrounding the epididymis. The absolute mass of this tissue in animals on HFD increased by almost four times compared with animals receiving an STD (1.7047 ± 0.4698 g in group 3 vs. 0.4556 ± 0.0708 g in group 1 and 0.4259 ± 0.0816 g in group 2). The same was observed for the relative mass of adipose tissue surrounding the epididymis (5.0842 ± 0.9595% in group 3 vs. 1.6562 ± 0.2532% in group 1 and 1.5005 ± 0.2371% in group 2). However, it should be noted that the absolute and relative masses of adipose tissue in the groups on HFD treated with AICAR (groups 4–6) were significantly reduced compared to group 3 (HFD + vehicle) (1.0991 ± 0.2389 g in the group 4, 1.2541 ± 0.3390 g in group 5, and 1.1489 ± 0.4382 g in group 6 vs. 1.7047 ± 0.4698 g in group 3 for absolute mass and 3.5412 ± 0.7005% in group 4, 4.1193 ± 0.8408% in group 5, and 3.6893 ± 1.0977% in group 6 vs. 5.0842 ± 0.9595% in group 3). Thus, the administration of AICAR, either alone or in combination with MXT, effectively reduced the adipose tissue mass in HFD-fed animals.

### 2.10. Histological Analysis Results

The summary data on the histological analysis are presented in Table 11. The microscopic analysis was carried out using an AxioScope. A1 transmitted light microscope (Carl Zeiss, Jena, Germany). The functional morphology of the liver of mice in control group 1, kept on STD, generally corresponded to the norm. At the same time, in five of the animals of this group, a few small foci of mononuclear infiltration were found in different parts of the organs. Against the background of the introduction of AICAR in male mice kept on STD (group 2), in nine cases, numerous small foci of mononuclear infiltration were noted and, in five animals, segmented leukocytes were also found in some of these foci and phenomena of phagocytosis of dead hepatocytes were observed. The described changes, apparently, are a manifestation of a mild hepatotoxic effect of AICAR. In addition, among the animals of this group in the liver, in three cases, focal hypertrophy of hepatocytes was observed. Under HFD conditions in animals from group 3, foci of mononuclear infiltration in the liver occurred in all the animals and segmented neutrophils in such foci were found in four cases. In half of the animals of the third group, a pronounced accumulation of glycogen was noted in hepatocytes, in two cases—lipid inclusions in the cytoplasm of liver cells. In the fourth group of animals, which were intraperitoneally injected with AICAR from the first week of the study, no lipid inclusions were found in the cytoplasm of hepatocytes and glycogen accumulation was observed in four cases (the total score for this feature 9 points in group 4 vs. 13 points in group 3). It should also be noted that the total estimated score of mononuclear infiltration in different lobes of the liver was high in group 2 vs. group 1 (score 14 points vs. 6 points), which may also indicate in the presence of AICAR a slight hepatotoxic effect. Lipid inclusions in the cytoplasm of hepatocytes most often (in five out of nine animals with a total score of 12 points) were found in group 5. In group 6 lipid inclusions were noted only in two cases with a total score of 4. At the same time, in group 4 no lipid inclusions were observed at all. Thus, the morphofunctional state of the liver in male mice kept on HFD was most favorable in group 6 (the lowest total score for all changes in the liver). The AICAR treatment from the first week (group 4) justified for the prevention of fatty hepatosis. However, it should be noted that in the absence of metabolic disorders in animals fed a standard diet, AICAR may exhibit some hepatotoxicity characterized by neutrophilic and mononuclear infiltration. In the pancreas of male mice of group 1, kept on STD, no abnormalities were noted. In group 2, foci of mononuclear interstitial infiltration were found in four male mice. In the metabolic syndrome model (HFD + vehicle), cell hyperplasia of the islets of Langerhans was observed in four males, foci of mononuclear infiltration of the gland stroma and focal vacuolar changes in the cells of the exocrine apparatus were observed in two and one cases, respectively. With the introduction of AICAR from the first week of the study (group 4), hyperplasia of the cells of the islet apparatus occurred in two animals. The introduction of AICAR from the seventh week of the study (group 5) alone or in combination with Methotrexate (group 6) did not contribute positive dynamics to the morphofunctional state of the pancreas compared to the control contained on HFD (group 3). On HFD, the state of the adrenal glands, compared with the control group of animals on a standard diet, did not become worse, with the exception of two cases of moderate focal hypertrophy of epithelial cells of the fascicular zone of the cortical substance in the group of animals treated with AICAR from the seventh week of the experiment (total assessment score 6 in the HFD + vehicle group versus 2 in the STD + vehicle group). In the kidneys of all animals of group 3, the initial manifestations of chronic progressive nephropathy were noted. In the animals treated with HFD, foci of mononuclear infiltration were observed in different parts of the organ; this change was most pronounced in animals from group 3—total score 14; group 4—6 points; group 5—3 points; and group 6—7 points versus 1 point in group 2 and 0 points in group 1. Prolonged exposure of rodents to a HFD led to severe irreversible changes in the renal parenchyma. Thus, dilatation of the renal pelvis (usually unilateral damage) with atrophy of the renal parenchyma was noted in five, four, seven, and five animals in the third, fourth, fifth, and sixth groups, respectively. Moreover, severe dilatation of the pelvis with total or subtotal atrophy of the renal parenchyma occurred in 5, 3, 1, and 4 cases, respectively. Thus, the most pronounced dilatation of the renal pelvis (piloectasia) was observed in group 3 (25 points); this disorder was least pronounced in animals treated with AICAR both from the first week (17 points) and from the seventh week (19 points). Along with pyelectasis in such animals, as a rule, numerous foci of mononuclear and neutrophilic infiltration were also found (the latter most often near the renal pelvis and ureter) and an expansion of the lumen of the renal tubules was noted. In all the animals treated with HFD, vacuolization of the cytoplasm of epitheliocytes was noted in the renal tubules. The total score for this trait in animals kept on HFD was minimal in the groups treated with AICAR (group 4—19 points and group 5—20 points) and the maximum—on the background of the introduction of saline (group 3 HFD + vehicle) (24 points). In the myocardium of all the male mice kept on HFD, lipid inclusions in the sarcoplasm of cardiomyocytes, as well as mineral deposits in the interstitium of the myocardium, were approximately equally observed. Thus, the histological study showed that keeping C57BL mice on a high-fat diet leads to changes characteristic of the metabolic syndrome with pronounced pathomorphological changes in the liver, kidneys, heart, and pancreas. The introduction of AICAR both as a monodrug and in combination with Methotrexate to correct the metabolic syndrome in all groups has a positive effect on the morphofunctional state of the liver.

**Figure 2:**
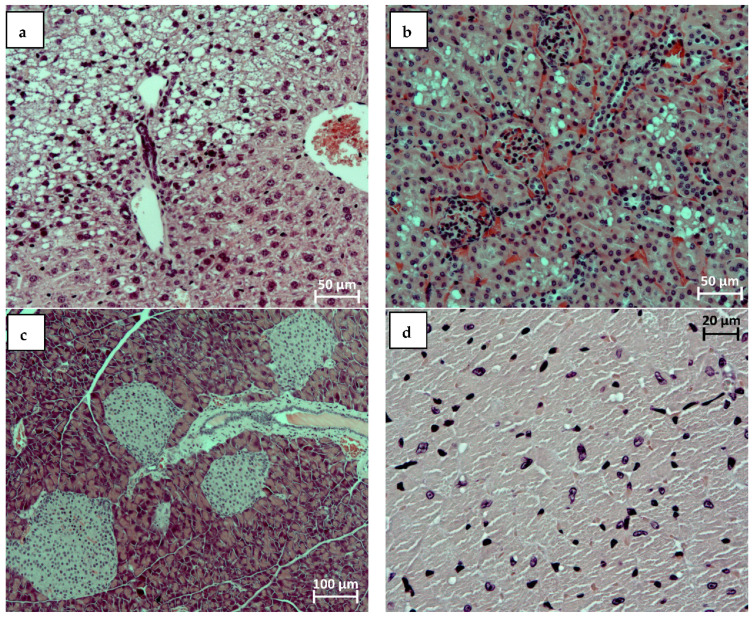
Fragments of the liver (**a**), kidneys (**b**), pancreas (**c**), and myocardium (**d**) of male C57Bl/6 mice kept on a HFD: focal large droplet fatty degeneration of hepatocytes (**a**); pronounced cytoplasm vacuolization of the epithelial cells of the renal tubules (**b**); hyperplasia of the cells of Langerhans islets (**c**); and small lipid inclusions in the sarcoplasm of left ventricle individual cardiomyocytes (**d**). Stained with hematoxylin and eosin. SW. 200× (**a**,**b**), 100× (**c**), and 50× (**d**).

Figure 2 shows micrographs of histological preparations, which display typical pathomorphological changes that develop in animals under conditions of HFD and are characteristic for metabolic syndrome manifestation.

## 3. Discussion

The AICAR dose of 500 mg/kg was chosen based on previous studies showing its positive effects in relation to the pathophysiological effects provoked in mice by maintenance on HFD (reduction in fatty hepatosis, reduction in kidney disease signs) [25,29]. The main goal of the study was to evaluate the effect of AICAR on the physiological parameters of the animals with metabolic syndrome and type 2 diabetes mellitus. These conditions were modeled by keeping the animals on 45% HFD in combination with keeping the animals under constant (24 h) light for 14 days from week 5 to week 7 of the study. The selected amount of fat in HFD, as well as the length of time animals were kept on HFD (13 weeks), was effective in modeling metabolic syndrome and type 2 diabetes, which is consistent with the results of numerous studies modeling these conditions [30,31,32], because the mechanisms of pathological conditions characteristic of the metabolic syndrome and type 2 diabetes mellitus caused by HFD overlap [33]. As a result, the animals developed metabolic syndrome and type 2 diabetes, which was confirmed by a change in the physiological parameters of the animals corresponding to the metabolic syndrome and type 2 diabetes. The C57BL/6 mouse strain is very sensitive to maintenance on HFD [34]. These animals gain weight more efficiently than other mouse models, including genetically modified ones [35]. This sensitivity may be due to the fact that the C57Bl/6J strain has a mutation in nicotinamide nucleotide transhydrogenase [36]. When C57BL/6 mice are kept on HFD for a long time, they show signs comparable to those of the metabolic syndrome in humans, which include obesity, insulin resistance, glucose intolerance, and metabolic abnormalities of biochemical parameters [37,38,39]. Thus, the animals showed an increase in body weight relative to the animals kept on an STD, starting from the fourth week of the study. Weight gain is an obligatory component of the development of the metabolic syndrome and is observed in all the studies that model the metabolic syndrome and type 2 diabetes by keeping animals on HFD [40,41]. Additionally, several studies have shown that circadian rhythm disturbances caused by constant exposure to light (which we used in our study) have an effect that aggravates the development of systemic metabolic disorders in the body [33,42]. Our goal was also to achieve a state comparable to type 2 diabetes (hyperglucosemia, hyperinsulinemia). The measurements of glucose and insulin levels were performed after an overnight fast. The model of diet-induced diabetes is representative, as has been shown in a number of studies [33,43]. The HFD-fed animals in our study developed signs of type 2 diabetes mellitus, such as elevated fasting glucose and insulin levels [30,40]. An approximately six-fold increase in HOMA-IR by week 12 in HFD-treated animals indicates the development of insulin resistance characteristic of metabolic syndrome and type 2 diabetes mellitus [40]. Chronic hyperglycemia is one of the main risk factors for the development of diabetic nephropathy [44]. The development of type 2 diabetes in animals with obesity due to HFD was also confirmed by the development of diabetic nephropathy, characterized by foci of mononuclear and neutrophilic infiltration, vacuolization of the cytoplasm of renal tubular epitheliocytes [45], and mononuclear infiltration of kidney tissues [46]. AICAR treatment led to a significant decrease in the number of foci of inflammatory cell infiltration, which is most likely due to the anti-inflammatory effect of AICAR. Thus, the anti-inflammatory activity of AICAR is manifested in the suppression of TNF-α induced expression of C3 (an acute phase of inflammation protein) in a dose dependent manner and the manifestation of this effect is not due to AMPK activation [47]. Changes in biochemical parameters indicate a metabolic disorder in animals treated with HFD. In particular, a decrease in the level of urea may indicate a reduced metabolism of amino acids, which may indicate liver pathology, which in turn is a consequence of obesity and fatty degeneration. An increase in cholesterol levels in the animals treated with HFD indicates a lipid metabolism disorder, also associated with the development of metabolic syndrome and diabetes in animals. Decreased alkaline phosphatase in HFD-treated animals indicate liver pathology [48]. In our study, AICAR reduced the cholesterol levels raised by keeping animals on HFD. AICAR has previously been shown to inhibit the synthesis of fatty acids and cholesterol in liver cells [9] and enhance fatty acid oxidation [10]. A decrease in blood albumin levels, and an associated decrease in the albumin/globulin ratio in animals treated with HFD, may also indicate liver pathology, namely increased protein loss, which may indicate the pathology of the kidneys and intestines [49]. In our study, the HFD-fed animals showed an increase in abdominal fat as well as an increase in the mass of fat surrounding the epididymis, which is a hallmark of visceral adiposity characteristic of metabolic syndrome and type 2 diabetes in animal models [31], as well as in people [50,51,52]. This pathological overgrowth of adipose tissue in HFD studies leads to the development of adipose tissue inflammation, including the secretion of adipokines and the activation of adipose resident macrophages, which has pathological consequences for all other organs and tissues [53]. Metabolic syndrome, provoked by a high-fat diet and accompanying obesity, is associated with visceral obesity, observed in our study in the form of a visual increase in abdominal fat and an increase in the mass of adipose tissue surrounding the epididimis [40]. AICAR treatment significantly reduced both the visually assessed amount of fat deposits in the abdominal cavity and also significantly reduced the mass of adipose tissue surrounding the epididymis. This effect was previously shown in a study of Giri et al. [41], where, in DIO mice, the amount of adipose tissue surrounding the seminal appendages was reduced by reducing the accumulation of fat in existing adipocytes by activating the expression of proliferator-activated receptor ɣ coactivator 1α (PGC1α), which is a coactivator of nuclear receptors and other transcription factors that controls oxidative metabolism in various tissues, including adipose [54,55]. In addition, the same work demonstrated the ability of AICAR to inhibit the differentiation of adipocytes in vitro [41]. Additionally, in the study, an indirect indication was obtained of a decrease in the level of anxiety, increased in the animals on HVD, both in our study and as previously shown [56], in the animals treated with AICAR from the first day of the study (HFD + AC 1). Thus, the study shows a correction of the signs of the metabolic syndrome and diabetes mellitus in the animals kept on HFD by the course administration of AICAR, which indicates its therapeutic potential for the treatment of these conditions.

## 4. Materials and Methods

### 4.1. Animals

All the procedures and manipulations with animals were approved by the Committee for Control over Care and Use of Laboratory Animals of BIBCh RAS (IACUC) (protocol number 696/19 from 18 February 2019) and were carried out in accordance with the EU Directive 2010/63/EU. In this study, 60 mature male mice of the C57BL/6J line, obtained from the Pushchino nursery of laboratory animals, were used. The animals were received at the age of 6 weeks, then adapted in the laboratory for 14 days. The animals with no signs of health deviations were selected for the experiment. The animals were kept in a barrier-type animal housing room with automatic change of day and night periods (08:00–20:00—“day”, 20:00–08:00—“night”) and at least a 12-fold change of the air volume of the room in hour. The standards for animal welfare are those defined by Directive 2010/63/EU on the protection of animals used for scientific purposes. SNIFF RI/M-H V1534-30 complete granular rodent food was autoclaved and fed ad libitum. A dust-free rodent bedding consisting of wood chips (LIGNOCEL BK8/15, JRS, Rosenberg, Germany) was used. Water was provided in plastic bottles ad libitum. Special houses were used to enrich the environment.

### 4.2. Groups and Doses

The animals were divided into groups so that the average weight did not differ between groups. The animals from groups 1 (receiving saline) and 2 (receiving AICAR) were kept on an STD. The animals from groups 3 (saline), 4 (AICAR from study week 1), 5 (AICAR from study week 7), and 6 (Methotrexate/AICAR from study week 7) were maintained on HFD (Table 12).

### 4.3. Test Articles

AICAR was produced by OKA-biotech, Russia using microbiological synthesis by cultivating *Bacillus subtilis* bacteria modified by the sequential introduction of mutations in a suitable nutrient medium according to the technology of the Federal Institution “State Research Institute of Genetics and Selection of Industrial Microorganisms of the National Research Center” “Kurchatov Institute”, Russia [57]. AICAR was provided to the laboratory in powder form. The doses for administration were prepared immediately prior to administration. The required amount of powder was weighed and dissolved in saline. The concentration of the resulting solution was 100 mg/mL, the volume of administration was 10 mL/kg, and the dose administered was 500 mg/kg.

The drug Methotrexat–Ebewe (Methotrexate, MTX) injection 10 mg/mL, Sandoz, Slovenia was diluted with saline to a concentration of 0.1 mg/mL; the injection volume was 10 mL/kg and the administered dose was 1 mg/kg.

### 4.4. Modeling Obesity, Metabolic Syndrome, and Diabetes Mellitus

Metabolic syndrome in animals was modeled by keeping the animals on a HFD for 13 weeks in combination with circadian rhythm disruption with two weeks of constant light (weeks 5 and 6 of the study) without a change of day and night in order to cause metabolic disorders, which will contribute to an increase in body weight [58].

### 4.5. Diet

#### 4.5.1. Standard Diet (STD)

The standard diet consisted of SNIFF RI/M-H V1534-30 complete granular rodent food containing 58% carbohydrates, 9% fat, 33% protein, and a calorific value of 306 kkal/100 g.

#### 4.5.2. High Fat Diet (HFD)

To prepare 1 kg of high-fat compound feed, 610 g of ground SNIFF compound feed and 360 g of rendered lard were prepared and 25 g of water at a temperature of 60–70 °C, 10 g of sodium chloride and 30 g of monosodium glutamate were added. The mixture was brewed to the consistency of dough and food granules were formed. Then, the granules were dried at a temperature of 60–70 °C for 10–12 h. The resulting mixture corresponded to a diet with a nutrient content of 45% fat, 35% carbohydrate, and 20% protein, with a total calorie content of 516 kkal/100 g. The prepared food was transferred to the territory where the animals were kept and distributed among the cages in accordance with the group affiliation. The prepared feed was stored at 4 °C for no more than 7 days.

### 4.6. Study Design

At the first stage of the study (weeks 1–6 of the study), AICAR was administered only to two groups: group 2—STD + AC 1 and group 4—HFD + AC 1 (Table 1). Starting from the 7th week of the study until the 12th week, AICAR as a monocomponent (group 5—HFD + AC 7) and is in combination with Methotrexate—an AMPK inhibitor that slows down the metabolism of AICAR in the cell (group 6—HFD + MTX + AC 7) [59]. Group 1, which received the STD and saline, served as the negative control, and group 3, which received the HFD and saline, served as the positive control. AICAR was administered intraperitoneally to animals from groups 2, 4, 5, and 6 three times a week at a dose of 500 mg/kg. Methotrexate was administered to animals from group 6 once a week intraperitoneally at a dose of 1 mg/kg (two hours before the administration of AICAR). The control animals from groups 1 and 3 were injected intraperitoneally with 0.9% sodium chloride solution (vehicle). The volume of administration was 10 mL/kg. After the introduction of drugs in animals, possible clinical signs of intoxication were recorded. Body weight was recorded weekly and feed intake was recorded once every two weeks. At the end of the study, the animals’ muscle strengths, motor activities, insulin resistances, and glucose tolerances were recorded. At the end of the study (week 13), the mice were euthanized (anesthetized with Zoletil:Xylazine followed by total blood sampling) and necropsied. Blood was extracted from the inferior vena cava for biochemical analysis and for the determination of insulin. Organs (kidneys, liver, adrenal glands, heart, pancreas, spleen) were examined for macrodamages and weighed. Particular attention was paid to the adipose tissue surrounding the epididymis as a sign of animal obesity. This adipose tissue was carefully removed and also weighed.

#### 4.6.1. Food Deprivation

The animals were deprived of food for 12 h before the glucose tolerance test and the test for insulin resistance, as well as before necropsy. Access to water was not restricted.

#### 4.6.2. Glucometry

Glucometry was performed to determine the level of glucose in the blood—a drop of blood was exctracted with a small incision in the tip of the tail and the glucose concentration was determined with a satellite^®^ Express glucometer. The manipulation was performed in the insulin resistance and glucose tolerance test.

#### 4.6.3. Insulin Resistance Test

The test was carried out on half of the animals from each group the day before the planned necropsy—the amount of glucose in the blood after an overnight fast was determined in dynamics 20, 40, 60, and 120 min after insulin administration (Insulin glulisine, Sanofi, subcutaneously, 2 IU/kg).

#### 4.6.4. Oral Glucose Tolerance Test

The test was performed on half of the animals from each group two days before the planned necropsy. The initial glucose level was measured in all the animals after an overnight fast, after which a 40% glucose solution was provided by gavage at a dose of 2 g/kg and the amount of glucose was measured 30, 60, 90, and 120 min after the glucose administration.

#### 4.6.5. Assessment of Motor Activity

The study of the locomotor activity of animals was carried out using the TSE Multi Conditioning System Extended Advanced. During 3 min of the animal being on the standard platform of the device, the following parameters were recorded: time of immobility, time of movement, total distance, time and distance in the center, time and distance on the periphery, speed of movement, number of visits to the center, and stands.

#### 4.6.6. Blood Samples

During necropsy, blood was extracted from all the animals for biochemical and enzyme-linked immunosorbent assays. Blood was extracted totally from the inferior vena cava after euthanasia of the animals by anesthesia with a Zoletil:Xylazine mixture into a test tube without anticoagulant and centrifuged after clotting to obtain serum. Serum from each animal was divided into two aliquots (one aliquot for analysis of biochemical parameters and insulin) and was immediately frozen at −20 °C until analysis.

#### 4.6.7. Blood Chemistry

The following parameters (Table 13) were determined in blood serum using a SAPPHIRE 400 automatic biochemical analyzer (Tokyo Boeki LTD, Tokyo, Japan) using Randox GB reagent kits appropriate for each parameter.

#### 4.6.8. Enzyme Immunoassay for the Determination of Insulin

Insulin was determined in the blood serum of all animals using non-competitive enzyme immunoassay with a test system (Insulin-ELISA-BEST, JSC Vector-BEST, Novosibirsk, Russia) and a Multiskan™ GO spectrophotometer (Termo Scientific, Waltham, MA, USA).

#### 4.6.9. Organ Weight

The following organs, shown in Table 14, were weighed for all the animals at scheduled necropsy. The paired organs were weighed together. The adipose tissue surrounding the epididymis was carefully removed during necropsy and weighed. In addition to the absolute mass of the organs, the percentage ratio of the mass of the organ to the body mass was determined immediately before necropsy.

#### 4.6.10. Histopathology

The histological analysis was performed for the following organs: kidneys, liver, adrenal glands, heart, and pancreas. The fixed tissues were dehydrated and impregnated with paraffin; sections were created from paraffin blocks. The sections were stained with standard hematoxylin and eosin. The sections were examined using light microscopy.

#### 4.6.11. Statistical Analysis

For all the quantitative data, descriptive statistics were used: mean values (MEAN) and standard deviation (SD) were calculated, which, together with the N value (number of variants in the group), are presented in the final tables. The normality of the variant in the groups was considered using the Shapiro–Wilk test at a 5% significance level. To establish between-group differences, the body weight and weight gain data were analyzed using ANOVA-2 multivariate analysis of variance followed by a Newman–Keuls or Duncan test. To establish intergroup differences in other indicators (feed intake, functional tests, biochemical parameters, organ weight) depending on the distribution, the data were analyzed using a one-way ANOVA analysis of variance or a Kruskal–Wallis test or pairwise comparison using the Mann–Whitney test. The statistical analysis was performed using STATISTICA 7.1 software. The differences were considered statistically significant at *p* ≤ 0.05.

## 5. Conclusions

To model the metabolic syndrome and type 2 diabetes, we effectively used a diet that contained 45% fat, 35% carbohydrate, and 20% protein. Starting from the fourth week, the body weight of the animals significantly increased relative to the animals on the standard diet, while the feed intake of these animals was reduced relative to the animals kept on STD. Glucometry performed during the life phase revealed an increase in glucose levels in animals kept on HFD, as well as a deterioration in glucose tolerance in the glucose tolerance test. The post-lifetime analyses confirmed that HFD-treated animals developed signs of metabolic syndrome and diabetes mellitus—increased levels of glucose, insulin, and cholesterol. The development of metabolic syndrome and diabetes was also confirmed by an increase in the HOMA-IR index in all the animals treated with HFD and pathological changes in biochemical parameters indicating a violation of lipid metabolism and liver function. At necropsy in the animals treated with HFD, a large number of fatty deposits in the abdominal cavity was revealed, as well as hydronephrosis of one of the kidneys. In the animals treated with HFD, there was a decrease in the mass of the liver, adrenal glands, and pancreas, as well as a significant increase in the mass of adipose tissue surrounding the epididymis. The introduction of AICAR, both alone and in combination with Methotrexate, reduced the body weight and body weight gain relative to animals on HFD, starting from the ninth week of the study. The feed intake in HFD-fed AICAR-treated animals was slightly higher than HFD-fed animals without treatment, indicating some normalization of this indicator. In the animals treated with AICAR from day 1 of the study, the fasting insulin levels did not differ significantly from the animals on STD, in contrast to the rest of the animals on HFD. In all the AICAR-treated animals, the visually assessed body fat was lower compared to untreated HFD animals. Additionally, all the animals treated with AICAR had a significantly reduced mass of adipose tissue surrounding the epididymis, relative to the animals on HFD without treatment. The histological analysis showed a mild hepatotoxic effect in AICAR used in STD animals. AICAR administered against the background of HDJ, on the contrary, improved the functional morphology of the liver—reduced the accumulation of glycogen in hepatocytes. AICAR, introduced from the first day of the study, reduced the content of lipid inclusions in the cytoplasm. Additionally, AICAR, administered against the background of HFD, improved the condition of the kidneys, impaired by HFD—reduced the foci of mononuclear infiltration in the kidneys and also reduced the dilatation of the renal pelvis, which was also confirmed by the number of cases of hydronephrosis (except for group 6—HFD + MXT + AK). Methotrexate administration had no effect on the therapeutic activity of AICAR in the study.

## Figures and Tables

**Table 1:** Body weight.

Week of the Study	Mean Body Weight, g					
	Group 1 STD + Vehicle	Group 2 STD + AC 1	Group 3 HFD + Vehicle	Group 4 HFD + AC 1	Group 5 HFD + AC 7	Group 6 HFD + MTX + AC 7
	n = 10	n = 10	n = 10	n = 10	n = 10 ^&^	n = 10 ^$^
0	21.4 ± 1.5	21.3 ± 1.7	21.7 ± 1.3	21.6 ± 1.2	21.7 ± 1.4	21.2 ± 2.1
1	23.2 ± 1.2	23.8 ± 1.1	23.4 ± 1.4	23.3 ± 1.6	22.8 ± 1.0	23.1 ± 1.3
2	24.1 ± 0.9	25.2 ± 1.5	24.2 ± 1.4	24.2 ± 1.9	23.6 ± 1.2	24.0 ± 1.4
3	24.1 ± 0.9	25.4 ± 1.7	24.6 ± 1.5	24.9 ± 1.9	24.1 ± 1.4	24.5 ± 1.3
4	24.7 ± 1.1	26.1 ± 1.5	26.8 ± 2.0 ***	25.9 ± 1.9	26.4 ± 1.7 *	26.7 ± 1.9 *
5	25.6 ± 1.1	27.0 ± 1.6	28.5 ± 2.2 ***	27.6 ± 2.2 *	28.5 ± 2.2 **	28.6 ± 2.4 **
6	25.7 ± 1.2	27.1 ± 1.6 @@	30.4 ± 2.7 ***	28.7 ± 2.0	30.3 ± 2.4 #	30.3 ± 3.0 #
7	26.1 ± 1.0	27.6 ± 1.6 @@	30.6 ± 2.6 ***	29.2 ± 2.1 **	29.2 ± 2.6 **	29.5 ± 2.6 ***
8	26.5 ± 1.1	27.9 ± 1.6 @@	30.5 ± 2.6 ***	29.0 ± 2.2 **	28.8 ± 2.1 *	29.2 ± 2.7 **
9	27.0 ± 1.0	28.2 ± 1.6 @@	31.7 ± 2.5 ***	29.9 ± 2.8 **	29.3 ± 2.4 *	29.2 ± 2.7 *@
10	27.0 ± 1.2	28.5 ± 1.5 @@@	32.2 ± 2.7 ***	30.8 ± 2.7 ***#	29.4 ± 2.4 **@	29.4 ± 2.8 *@@
11	27.5 ± 1.2	28.6 ± 1.5 @@@	32.9 ± 3.0 ***	30.9 ± 2.9 **#	29.9 ± 2.2 *@	29.6 ± 3.0 @
12	27.3 ± 1.3	29.0 ± 1.3 @@@	33.6 ± 3.3 ***	31.7 ± 3.0 ***#	30.4 ± 2.4 **@	30.3 ± 3.3 **@@

^&^—n = 9 starting from the 10th week due to the death of one animal; ^$^—n = 9 starting from the 9th week due to the death of one animal; * *p* ≤ 0.05, ** *p* ≤ 0.01, *** *p* ≤ 0.001 relative to group 1 (STD + vehicle) according to ANOVA 2; @ *p* ≤ 0.05, @@ *p* ≤ 0.01, @@@ *p* ≤ 0.001 relative to group 3 (HFD + vehicle) according to ANOVA 2; # *p* ≤ 0.05 relative to group 2 (STD + AC 1) according to ANOVA 2.

**Table 2:** Weight gain.

Week of the Study	Average Weight Gain Relative to the First Day of the Study, %					
	Group 1 STD + vehicle	Group 2 STD + AC 1	Group 3 HFD + vehicle	Group 4 HFD + AC 1	Group 5 HFD + AC 7	Group 6 HFD + MTX + AC 7
	n = 10	n = 10	n = 10	n = 10	n = 10 ^&^	n = 10 ^$^
1	8.8 ± 5.0	12.2 ± 7.0	8.0 ± 5.7	7.8 ± 7.6	5.1 ± 4.5	9.2 ± 6.2
2	13.1 ± 6.2	19.0 ± 12.1	11.7 ± 5.5	12.2 ± 10.9	8.9 ± 5.0	13.5 ± 7.0
3	13.5 ± 7.7	19.8 ± 12.8	13.9 ± 6.3	15.3 ± 11.2	11.6 ± 7.3	15.7 ± 6.7
4	16.2 ± 7.7	23.0 ± 12.5	24.0 ± 8.0	20.1 ± 11.	22.0 ± 7.8	26.1 ± 5.9 *
5	20.1 ± 8.4	27.3 ± 13.7	31.6 ± 9.2 *	28.0 ± 13.9	31.7 ± 10.5 *	35.2 ± 8.8 **
6	20.7 ± 8.3	28.1 ± 13.6 @	40.5 ± 11.2 ***	33.1 ± 12.2 *	40.1 ± 11.2 ***#	42.9 ± 10.5 ***#
7	22.6 ± 6.9	30.4 ± 14.0 @	41.7 ± 12.4 ***	35.3 ± 13.6 *	35.2 ± 14.2 *	39.6 ± 11.1 **
8	24.3 ± 8.7	31.7 ± 14.4	41.3 ± 12.8 **	34.3 ± 13.5	33.1 ± 12.1	38.6 ± 10.5 **
9	26.7 ± 9.0	32.9 ± 13.6 @	46.6 ± 12.6 ***	38.7 ± 16.7 *	35.2 ± 11.9	38.6 ± 13.0
10	26.6 ± 8.5	34.4 ± 14.3 @@	49.0 ± 13.2 ***	42.8 ± 16.4 **	36.0 ± 12.8	39.8 ± 14.0 *
11	29.4 ± 8.4	34.9 ± 14.1 @@	52.0 ± 14.9 ***	43.1 ± 16.6 *	38.6 ± 12.7 @	40.6 ± 13.0
12	28.0 ± 6.0	36.8 ± 13.0 @@	55.6 ± 15.7 ***	47.2 ± 17.8 **	40.8 ± 13.8 *	43.4 ± 11.8 *

^&^—n = 9 starting from the 10th week due to the death of one animal; ^$^—n = 9 starting from the 9th week due to the death of one animal; * *p* ≤ 0.05, ** *p* ≤ 0.01, *** *p* ≤ 0.001 relative to group 1 (STD + vehicle) according to ANOVA 2; @ *p* ≤ 0.05, @@ *p* ≤ 0.01 relative to group 3 (HFD + vehicle) according to ANOVA 2; # *p* ≤ 0.05 relative to group 2 (STD + AC 1) according to ANOVA 2.

**Table 3:** Mean values of glucose concentration in the insulin resistance test.

Group	Group 1 STD + Vehicle	Group 2 STD + AC 1	Group 3 HFD + Vehicle	Group 4 HFD + AC 1	Group 5 HFD + AC 7	Group 6 HFD + MTX + AC 7
	n = 5	n = 5	n = 5	n = 5	n = 4 ^&^	n = 4 ^$^
Initial values, mmol/L	5.9 ± 1.5	6.9 ± 1.1	7.8 ± 2.2	8.9 ± 1.5 **##	8.5 ± 1.3 **	9.1 ± 0.7 **##
After 20 min, mmol/L	4.1 ± 0.3 ^	3.5 ± 0.5 ^^^	5.2 ± 1.0 *##^^	6.1 ± 1.1 ***###^^^	4.9 ± 0.6 #^^^	6.2 ± 0.7 ***###^^^
After 40 min, mmol/L	3.1 ± 07 ^^^	3.1 ± 0.4 ^^^	4.6 ± 0.4 **##^^	3.7 ± 0.6 ^^^	3.4 ± 0.4 @^^^	4.1 ± 1.5^^^
After 60 min, mmol/L	2.7 ± 1.0 ^^^	2.7 ± 0.7 ^^^	3.6 ± 04 *#^^^	3.5 ± 0.7 ^^^	3.0 ± 0.3 ^^^	2.8 ± 0.4 ^^^
After 120 min, mmol/L	3.2 ± 1.6 ^^^	2.1 ± 1.0 ^^^	3.6 ± 1.4 ^^^	3.3 ± 0.5 ^^^	2.9 ± 0.4 ^^^	3.5 ± 0.5 ^^^

^&^—n = 9 starting from the 10th week due to the death of one animal; ^$^—n = 9 starting from the 9th week due to the death of one animal; * *p* ≤ 0.05, ** *p* ≤ 0.01, *** *p* ≤ 0.001 relative to group 1 (STD + vehicle) according to one way ANOVA 1; # *p* ≤ 0.05, ## *p* ≤ 0.01, ### *p* ≤ 0.001 relative to group 2 (STD + AK 1) according to one way ANOVA 1; @ *p* ≤ 0.05 relative to group 3 (permit + carrier) according to one way ANOVA 1; ^ *p* ≤ 0.05 ^^ *p* ≤ 0.01, ^^^ *p* ≤ 0.001 relative to baseline (ANOVA 2).

**Table 4:** Food consumption.

Week of the Study	Mean Food Consumption, %					
	Group 1 STD + Vehicle	Group 2 STD + AC 1	Group 3 HFD + Vehicle	Group 4 HFD + AC 1	Group 5 HFD + AC 7	Group 6 HFD + MTX + AC 7
	n = 10	n = 10	n = 10	n = 10	n = 10 ^&^	n = 10 ^$^
1	171.1 ± 14.8	161.3 ± 16.8	104.9 ± 21.1 ***###	103.1 ± 31.1 ***###	69.5 ± 31.2 ***###	91.8 ± 25.4 ***###
3	191.0 ± 41.2	194.9 ± 27.6	130.0 ± 20.0 ***###	118.7 ± 30.4 ***###	120.9 ± 9.3 ***###	135.1 ± 16.9 ***###
5	156.6 ± 14.5 ^^%%%	162.6 ± 12.0 ^^%%	104.8 ± 11.2 ***###^^^%%%ββ	124.2 ± 27.2 ***###@@	110.8 ± 18.5 ***###%®®®	112.1 ± 11.6 ***###^%
7	196.3 ± 19.3 %	206.8 ± 31.2 ^^%%	128.9 ± 7.1 ***###^^^%%%	121.4 ± 29.0 ***###	128.2 ± 12.9 ***###§^^^%%%®®	132.3 ± 10.1 ***###^^^%%%
9	143.6 ± 13.1 ^^^%®®	160.2 ± 29.6 ^^^%%%	82.8 ± 10.1 ***###^^^®	102.1 ± 21.1 ***###	98.5 ± 20.1 ***###^%%%	106.0 ± 29.8 ***###@^^%
11	138.7 ± 33.2 ^^^%®	143.8 ± 20.5 ^^^%%%	118.4 ± 16.2 #^^^βββ	122.1 ± 17.8 #	115.3 ± 16.2 *#®®®	97.8 ± 31.8 ***###§^^^%%

^&^—n = 9 starting from the 10th week due to the death of one animal; ^$^—n = 9 starting from the 9th week due to the death of one animal; * *p* ≤ 0.05, *** *p* ≤ 0.001 relative to group 1 (STD + vehicle) according to ANOVA 2; @ *p* ≤ 0.05, @@ *p* ≤ 0.01 relative to group 3 (HFD + vehicle) according to ANOVA 2; # *p* ≤ 0.05, ### *p* ≤ 0.001 relative to group 2 (STD + AC 1) according to ANOVA 2; § *p* ≤ 0.05 relative to group 4 (STD + AC 1) according to ANOVA 2; ^ *p* ≤ 0.05, ^^ *p* ≤ 0.01, ^^^ *p* ≤ 0.001 relative to week 1 according to ANOVA 1; % *p* ≤ 0.05, %% *p* ≤ 0.01, %%% *p* ≤ 0.001 relative to week 3 according to ANOVA 1; ® *p* ≤ 0.05, ®® *p* ≤ 0.01, ®®® *p* ≤ 0.001 relative to week 7 according to ANOVA 1; ββ *p* ≤ 0.01, βββ *p* ≤ 0.001 relative to week 9 according to ANOVA 1.

**Table 5:** Mean fasting insulin and glucose values on the day of euthanasia.

Group	Group 1 STD + Vehicle	Group 2 STD + AC 1	Group 3 HFD + Vehicle	Group 4 HFD + AC 1	Group 5 HFD + AC 7	Group 6 HFD + MTX + AC 7
	n = 10	n = 10	n = 10	n = 10	n = 9 ^&^	n = 9 ^$^
Insulin, mIU/L	6.23 ± 5.20	7.82 ± 4.10	32.92 ± 15.09 *	18.31 ± 9.50 @	19.58 ± 6.74 *@	21.81 ± 11.36 *
Glucose, mmol/L	6.05 ± 1.18	6.52 ± 1.03	7.63 ± 1.78 €	8.36 ± 1.65 *	8.44 ± 0.88 *	8.7 ± 11.36 *

^&^—n = 9 starting from the 10th week due to the death of one animal; ^$^—n = 9 starting from the 9th week due to the death of one animal; * *p* ≤ 0.05 regarding group 1 according to Kruskal—Walles ANOVA; @ *p* ≤ 0.05 regarding group 3 according to Mann–Whitney U test; € *p* ≤ 0.05 regarding group 1 according to Mann–Whitney U test.

**Table 6:** Mean values of glucose concentration in the oral glucose tolerance test.

Group	Group 1 STD + Vehicle	Group 2 STD + AC 1	Group 3 HFD + Vehicle	Group 4 HFD + AC 1	Group 5 HFD + AC 7	Group 6 HFD + MTX + AC 7
	n = 5	n = 5	n = 5	n = 5	n = 5	n = 5
Initial values, mmol/L	5.2 ± 0.4	6.2 ± 0.9	7.4 ± 1.5 **	7.9 ± 1.8 ***##	8.4 ± 0.6 ***##	7.4 ± 0.9 **
After 30 min, mmol/L	12.0 ± 1.0 βββ	12.1 ± 1.6 βββ	13.0 ± 1.7 βββ	13.7 ± 1.0 βββ	14.9 ± 2.7 **##βββ	14.7 ± 1.0 *#βββ
After 60 min, mmol/L	8.2 ± 1.7 βββψψψ	7.9 ± 1.2 βψψψ	10.1 ± 1.8 ##ββψψψ	10.7 ± 1.5 *##ββψψ	11.5 ± 1.0 **##ββψψ	11.0 ± 1.8 **##βββψψψ
After 90 min, mmol/L	6.9 ± 0.5 ββψψψ	7.3 ± 0.8 ψψψ	8.4 ± 1.2 *ψψ	9.3 ± 0.8 ***##ψψψ	10.2 ± 0.3 ***###@@ψψψ	10.1 ± 1.4 ***###@@ββψψψ
After 120 min, mmol/L	6.3 ± 0.7 ψψψ	8.5 ± 1.3 *ββψψψ	10.2 ± 1.4 ***ββψψ	9.8 ± 1.8 ***ψψψ	11.5 ± 1.9 ***##ββψψ	10.0 ± 1.1 ***ββψψψ

* *p* ≤ 0.05, ** *p* ≤ 0.01, *** *p* ≤ 0.001 relative to group 1 (one way ANOVA); # *p* ≤ 0.05, ## *p* ≤ 0.01, ### *p* ≤ 0.001 relative to group 2 (one way ANOVA); @@ *p* ≤ 0.01 relative to group 3 (one way ANOVA); β *p* ≤ 0.05, ββ *p* ≤ 0.01, βββ *p* ≤ 0.001 relative to baseline, (ANOVA 2); ψψ *p* ≤ 0.01, ψψψ *p* ≤ 0.001 relative to the 30th minute, (ANOVA 2).

**Table 7:** Results of biochemical analysis of animal blood plasma.

Group	Group 1 STD + Vehicle	Group 2 STD + AC 1	Group 3 HFD + Vehicle	Group 4 HFD + AC 1	Group 5 HFD + AC 7	Group 6 HFD + MTX + AC 7
	n = 10	n = 10	n = 10	n = 10	n = 9 ^&^	n = 9 ^$^
Urea, mmol/L	10.7 ± 0.8	9.5 ± 1.0	6.9 ± 0.7 *	6.7 ± 1.0 **	6.0 ± 0.5 ***##	6.2 ± 0.9 ***##
Cholesterol, mmol/L	2.68 ± 0.34	2.28 ± 0.42	5.50 ± 0.69 ***###	4.67 ± 0.52 *##@@	4.71 ± 0.38 *#@	4.53 ± 0.43 #@@
Triglycerides, mmol/L	1.33 ± 0.25	1.04 ± 0.24	1.62 ± 0.64	1.02 ± 0.25	1.20 ± 0.15	0.97 ± 0.15 *@
ALT, U/L	63 ± 8	54 ± 13	37 ± 7 **	35 ± 10 ***#	35 ± 7 ***#	31 ± 4 ***##
AST, U/L	67 ± 6	61 ± 13	57 ± 4	57 ± 8	58 ± 6	53 ± 4 **
Total bilirubin, mmol/L	7.6 ± 1.6	8.2 ± 1.1	6.4 ± 2.6	6.5 ± 1.8	5.1 ± 1.8 ##	6.6 ± 1.6
Creatinine, mmol/L	48 ± 7	48 ± 4	47 ± 4	46 ± 4	46 ± 8	41 ± 4
Alkaline phosphatase, U/L	98 ± 9	88 ± 11	54 ± 7 ***##	54 ± 8 **#	54 ± 8 **#	50 ± 5 ***###
Albumen, g/L	41.9 ± 2.2	39.0 ± 2.1	40.6 ± 1.7	38.8 ± 1.4 *	39.8 ± 2.0	38.4 ± 2.0 *
Calcium, mmol/L	2.86 ± 0.19	2.72 ± 0.13	2.84 ± 0.14	2.75 ± 0.08	2.81 ± 0.15	2.66 ± 0.11
Phosphates, mmol/L	3.94 ± 0.53	3.63 ± 0.34	3.59 ± 0.50	3.62 ± 0.34	3.60 ± 0.39	3.28 ± 0.17 *
Total protein, g/L	65.1 ± 3.9	61.7 ± 2.9	65.6 ± 2.7	63.1 ± 3.3	63.2 ± 4,0	61.7 ± 3.2
Chlorides, mmol/L	127.1 ± 3.5	126.0 ± 2.1	130.4 ± 4.1	128.6 ± 7.8	126 ± 3	124 ± 2 @
Globulin, g/L	23.1 ± 2.1	22.6 ± 1.0	25.0 ± 1.5	24.3 ± 2.3	23.4 ± 2.4	23.3 ± 1.5
Albumin/globulin	1.8 ± 0.1	1.7 ± 0.1	1.6 ± 0.1 **	1.6 ± 0.1 **	1.7 ± 0.1	1.7 ± 0.1 *

^&^—n = 9 starting from the 10th week due to the death of one animal; ^$^—n = 9 starting from the 9th week due to the death of one animal; * *p* ≤ 0.05, ** *p* ≤ 0.01, *** *p* ≤ 0.001 relative to group 1 (Kruskal–Wallis ANOVA); # *p* ≤ 0.05, ## *p* ≤ 0.01, ### *p* ≤ 0.001 relative to group 2 (Kruskal–Wallis ANOVA); @ *p* ≤ 0.05, @@ *p* ≤ 0.01 relative to group 3 (Mann–Whitney U test).

**Table 8:** The results of testing the motor activity of animals.

Parameter	Group 1 STD + Vehicle	Group 2 STD + AC 1	Group 3 HFD + Vehicle	Group 4 HFD + AC 1	Group 5 HFD + AC 7	Group 6 HFD + MTX + AC 7
	n = 10	n = 10	n = 10	n = 10	n = 9 ^&^	n = 9 ^$^
Resting time (s)	105 ± 19	105 ± 23	109 ± 21	99 ± 11	101 ± 29	101 ± 12
Moving time (s)	75 ± 19	75 ± 23	71 ± 21	81 ± 11	79 ± 29	79 ± 12
Distance (m)	16 ± 4	17 ± 5	14 ± 5	16 ± 2	16 ± 6	16 ± 3
Time in center (s)	53 ± 54	61 ± 52	32 ± 28	39 ± 28	32 ± 43	39 ± 41
Distance in center (m)	4 ± 3	5 ± 1	2 ± 1 ##	3 ± 2	2 ± 1 #	2 ± 2 #
Periphery time (s)	127 ± 54	119 ± 52	148 ± 28	141 ± 38	148 ± 43	141 ± 41
Periphery distance (m)	12 ± 5	12 ± 6	12 ± 5	13 ±3	14 ± 6	14 ± 4
Visits to center	12 ± 7	15 ± 8	10 ± 10	12 ± 12	9 ± 6	6 ± 5
Rearings	5 ± 5	5 ± 7	6 ± 8	6 ± 6	5 ± 5	7 ± 7

^&^—n = 9 starting from the 10th week due to the death of one animal; ^$^—n = 9 starting from the 9th week due to the death of one animal; # *p* ≤ 0,05, ## *p* ≤ 0,01 relative to group 2 (STD + AC) according to ANOVA 1.

**Table 9:** Macroscopic abnormalities detected in animals during necropsy.

Deviation from the Norm	The Number of Animals with a Sign of Deviation from the Norm					
	Group 1 STD + Vehicle	Group 2 STD + AC 1	Group 3 HFD + Vehicle	Group 4 HFD + AC 1	Group 5 HFD + AC 7	Group 6 HFD + MTX + AC 7
	n = 10	n = 10	n = 10	n = 10	n = 9 ^&^	n = 9 ^$^
No macroscopic abnormalities were found	10	10	0 ***	3 ***	4 **@	1 ***
Visually in the abdominal cavity a large amount of fat deposits	0	0	10 ***	4 *@	4 *@@	5 **@
Hydronephrosis of the kidney	0	0	5 **	3	1	4 *
Enlargement of the spleen	0	0	1	1	0	0

^&^—n = 9 starting from the 10th week due to the death of one animal; ^$^—n = 9 starting from the 9th week due to the death of one animal; * *p* ≤ 0.05, ** *p* ≤ 0.01, *** *p* ≤ 0.001 relative to group 1 according to Chi-square test; @ *p* ≤ 0.05, @@ *p* ≤ 0.01 relative to group 3 according to Chi-square test.

**Table 10:** Organ mass in animals.

Organ	Group 1 STD + Vehicle	Group 2 STD + AC 1	Group 3 HFD + Vehicle	Group 4 HFD + AC 1	Group 5 HFD + AC 7	Group 6 HFD + MTX + AC 7
	n = 10	n = 10	n = 10	n = 10	n = 9 ^&^	n = 9 ^$^
Absolute mass of organs, g						
Testes, g.	0.187 ± 0.023	0.196 ± 0.015	0.188 ± 0.014	0.192 ± 0.019	0.188 ± 0.019	0.180 ± 0.017
Spleen, g	0.084 ± 0.037	0.089 ± 0.020	0.105 ± 0.043	0.201 ± 0.279	0.103 ± 0.039	0.081 ± 0.016
Kidneys, g	0.484 ± 0.390	0.433 ± 0.060	0.575 ± 0.186	0.465 ± 0.128	0.428 ± 0.157	0.440 ± 0.121
Adrenal gland, g.	0.0079 ± 0.0011	0.0075 ± 0.0025	0.0088 ± 0.0020	0.0144 ± 0.0243	0.0038 ± 0.0010 *#@@@	0.0048 ± 0.0021 @@
Liver, g.	1.59 ± 0.43	1.73 ± 0.19	1.37 ± 0.18	1.28 ± 0.19 *##	1.27 ± 0.10 *##	1.28 ± 0.14 *##
Heart, g.	0.146 ± 0.020	0.146 ± 0.019	0.159 ± 0.015	0.158 ± 0.028	0.151 ± 0.014	0.148 ± 0.012
Pancreas, g.	0.158 ± 0.017	0.220 ± 0.075	0.160 ± 0.027	0.236 ± 0.272	0.118 ± 0.017 *###@@	0.148 ± 0.047 ##
Adipose tissue surrounding the epididymis, g.	0.4556 ± 0.0708	0.4259 ± 0.0816	1.7047 ± 0.4698 ***###	1.0991 ± 0.2389 #∞∞∞	1.2541 ± 0.3390 **#∞	1.1489 ± 0.4382 *##∞
Relative mass of organs (relative to body mass), %						
Testes, %	0.679 ± 0.077	0.693 ± 0.061	0.571 ± 0.068 *##	0.622 ± 0.074	0.626 ± 0.051	0.597 ± 0.040
Spleen, %	0.304 ± 0.131	0.315 ± 0.064	0.325 ± 0.169	0.682 ± 1.039	0.351 ± 0.159	0.269 ± 0.065
Kidneys, %	1.783 ± 1.495	1.526 ± 0.142	1.749 ± 0.591	1.504 ± 0.419	1.428 ± 0.517	1.453 ± 0.430
Adrenal gland, %.	0.0288 ± 0.0041	0.0264 ± 0.0075	0.0272 ± 0.0056	0.0438 ± 0.0693	0.0126 ± 0.0030 ***##@@@	0.0157 ± 0.0072 **@@
Liver, %.	5.752 ± 1.526	6.124 ± 0.681	4.141 ± 0.458 **#	4.148 ± 0.502 *##	4.253 ± 0.485 *##	4.199 ± 0.333 ##
Heart, %.	0.528 ± 0.059	0.516 ± 0.058	0.483 ± 0.062	0.508 ± 0.056	0.505 ± 0.073	0.486 ± 0.038
Pancreas, g.	0.575 ± 0.061	0.771 ± 0.234	0.484 ± 0.077	0.782 ± 0.954	0.393 ± 0.041 ***###	0.499 ± 0.211 ##
Adipose tissue surrounding the epididymis, %.	1.6562 ± 0.2532	1.5005 ± 0.2371	5.0842 ± 0.9595 ***###	3.5412 ± 0.7005 #∞∞∞	4.1193 ± 0.8408 ***##∞	3.6893 ± 1.0977 *##∞

^&^—n = 9 starting from the 10th week due to the death of one animal; ^$^—n = 9 starting from the 9th week due to the death of one animal; * *p* ≤ 0.05, ** *p* ≤ 0.01, *** *p* ≤ 0.001 comparing to group 1 (Kruskal–Wallis ANOVA); # *p* ≤ 0.05, ## *p* ≤ 0.01, ### *p* ≤ 0.001 comparing to group 2 (Kruskal–Wallis ANOVA); @@ *p* ≤ 0.01, @@@ *p* ≤ 0.001 comparing to group 3 (Kruskal–Wallis ANOVA); ∞ *p* ≤ 0.05, ∞∞∞ *p* ≤ 0.001 comparing to group 3 (Mann–Whitney U test).

**Table 11:** Deviations from the norm revealed during microscopic analysis in organs and tissues.

	Group 1 STD + Vehicle	Group 2 STD + AC 1	Group 3 HFD + Vehicle	Group 4 HFD + AC 1	Group 5 HFD + AC 7	Group 6 HFD + MTX + AC 7
Animals Number	n = 10	n = 10	n = 10	n = 10	n = 9	n = 9
Description of changes in organs	The number and severity of changes in the organs					
LIVER:						
Hypertrophy of hepatocytes	0	3SL	1SL	1SL	0	0
TOTAL SCORE	0	6	2	1	0	0
Foci of mononuclear infiltration	4MI 1SL	4MI 5SL	4MI 6SL	1MI 9SL	2MI 6SL	2MI 4SL
TOTAL SCORE	6	14	16	19	14	10
Segmented neutrophils in foci of mononuclear infiltration	0	3MI 2SL	4SL	4SL	1MI 2SL	2SL
TOTAL SCORE	0	7	8	8	5	4
Focal angiectasia and intrahepatocellular erythrocytes	0	1SL	0	0	1SL	0
TOTAL SCORE	0	2	0	0	2	0
Accumulation of glycogen in hepatocytes	0	0	2SL 3MO	3SL 1MO	4SL	2SL 1MO
TOTAL SCORE	0	0	13	9	8	7
Lipid inclusions in the cytoplasm of hepatocytes	0	0	1SL 1MO	0	1MI 2SL 1MO 1MA	1MI 1MO
TOTAL SCORE	0	0	5	0	12	4
Description of changes in organs	The number and severity of changes in the organs					
PANCREAS:						
Cell hyperplasia of the islets of Langerhans	0	1SL	4SL	1SL 1MO	3SL 1MO	2SL 2MO
TOTAL SCORE	0	2	8	5	9	10
Foci of mononuclear infiltration	0	1MI 2SL 1MO	1MI 1SL	1SL	1SL	1MI 1SL
TOTAL SCORE	0	8	3	2	2	3
Lipomatosis	3MI	2MI	1MI 1SL	3MI	3MI	1MI 1SL
TOTAL SCORE	3	2	3	3	3	3
Exocrinocytes with vacuolar changes in the cytoplasm	0	1MI	1MI	0	0	0
TOTAL SCORE	0	1	1	0	0	0
TOTAL SCORE	0	1	1	0	0	0
ADRENALS:						
Hypertrophy of epithelial cells of the fascicular zone of the cortical substance	1SL	0	1MI	1SL	2MO	0
TOTAL SCORE	2	0	1	2	6	0
KIDNEYS:						
Chronic progressive nephropathy	2MI	1MI 1SL	2SL	1MI 1SL	1MI 1SL 1MO	1MI 1SL
TOTAL SCORE	2	3	4	3	6	3
Foci of mononuclear infiltration	0	1MI	1MI 1SL 1MO 2MA	2MO	1MO	1MI 2MO
TOTAL SCORE	0	1	14	6	3	7
Foci of accumulation of siderophages	0	0	1MI	1MI	0	1MI
TOTAL SCORE	0	0	1	1	0	1
	Group 1 STD + vehicle	Group 2 STD + AC 1	Group 3 HFD + vehicle	Group 4 HFD + AC 1	Group 5 HFD + AC 7	Group 6 HFD + MTX + AC 7
Animals number	n = 10	n = 10	n = 10	n = 10	n = 9	n = 9
Description of changes in organs	The number and severity of changes in the organs					
Foci of neutrophilic infiltration	0	0	1SL 1MO	2SL 1MO	1SL	2SL 1MO
TOTAL SCORE	0	0	5	7	2	7
Accumulation of neutrophils in the lumen of the ureter	0	0	0	1MO	0	0
TOTAL SCORE	0	0	0	3	0	0
Vacuolization of the cytoplasm of renal tubular epithelial cells	0	1MI	2MI 1SL 6MO 1MA	3MI 3SL 2MO 1MA	1MI 5SL 3MO	1MI 3SL 4MO 1MA
TOTAL SCORE	0	1	24	19	20	23
Dilatation of the renal pelvis	0	0	5SE	1SL 3SE	4SL 2MO 1SE	1SL 2MA 2SE
TOTAL SCORE	0	0	25	17	19	20
Atrophy of the renal parenchyma	0	0	2MA 3SE	1SL 3MA	4SL 2MO 1SE	1SL 2MO 1MA 1SE
TOTAL SCORE	0	0	23	14	19	17
Expansion of the lumen of the renal tubules	0	0	1SL 3MO	3MO	2SL 1MO	1SL 2MO 1MA
TOTAL SCORE	0	0	11	9	7	12
HEART:						
Mineral deposits in myocardial interstitium	0	0	1MI	1MI 1SL	1MI 2SL	1MI
TOTAL SCORE	0	0	1	3	5	1
Lipid inclusions in the sarcoplasm of cardiomyocytes	0	0	3SL	3MI 1SL	4MI 1SL	3MI 1SL
TOTAL SCORE	0	0	6	5	6	5

Severity grading scale: MI—Minimal-1 score; SL—Slight-2 score; MO—Moderate-3 score; MA—Marked-4 score; SE—Severe-5 score.

**Table 12:** Groups and doses.

Group #	Diet	Group	Articles, Doses, Scheme	Animal Numbers
1	STD	STD + vehicle	Saline	1–10
2	STD	STD + AC 1	AICAR (500 mg/kg/three times a week from study week 1, 10 mL/kg, ip)	11–20
3	HFD	HFD + vehicle	Saline (three times a week from study week 1, 10 mL/kg, ip)	21–30
4	HFD	HFD + AC 1	AICAR (500 mg/kg/three times a week from the 1st week of the study, 10 mL/kg, ip)	31–40
5	HFD	HFD + AC 7	AICAR (500 mg/kg/three times a week from the 7th week of the study, 10 mL/kg, ip)	41–50
6	HFD	HFD + MTX + AC 7	Metotrexate + AICAR (1 mg/kg/weekly 10 mL/kg, ip + 500 mg/kg/three times a week from the 7th week of the study, 10 mL/kg, ip)	51–60

STD—standard diet; HFD—high-fat diet; AC—AICAR; MTX—Metotrexate; ip—intraperitoneally.

**Table 13:** Clinical biochemistry parameters.

Alanine transaminase (alt)	Calcium	Urea
Aspartate aminotransferase (ast)	Chloride	Total bilirubin
Alkaline phosphatase (ap)	Phosphate	Albumin
Creatinine	Cholesterine	Globulin
Total protein	Triglyceride	Albumin/globulin *

* Calculated parameter.

**Table 14:** Weighed Organs.

Adrenal gland	Kidneys
Heart	Spleen
Pancreas	Testes
Liver	Adipose tissue surrounding the epididymis

## Data Availability

All data is available upon request by e-mail to the corresponding author.
